# Clam Shell-Derived Hydroxyapatite: A Green Approach for the Photocatalytic Degradation of a Model Pollutant from the Textile Industry

**DOI:** 10.3390/ma17112492

**Published:** 2024-05-22

**Authors:** Roxana Ioana Matei (Brazdis), Anda Maria Baroi, Toma Fistos, Irina Fierascu, Maria Grapin, Valentin Raditoiu, Florentina Monica Raduly, Cristian Andi Nicolae, Radu Claudiu Fierascu

**Affiliations:** 1 National Institute for Research & Development in Chemistry and Petrochemistry—ICECHIM Bucharest, 202 Spl. Independentei, 060021 Bucharest, Romania; roxana.brazdis@icechim.ro (R.I.M.); anda.baroi@icechim.ro (A.M.B.); toma.fistos@icechim.ro (T.F.); irina.fierascu@icechim.ro (I.F.); maria.grapin@icechim.ro (M.G.); vraditoiu@icechim.ro (V.R.); monica.raduly@icechim.ro (F.M.R.); cristian.nicolae@icechim.ro (C.A.N.); 2 Faculty of Chemical Engineering and Biotechnologies, National University of Science and Technology Politehnica Bucharest, 1-7 Gh. Polizu Str., 011061 Bucharest, Romania; 3 Faculty of Horticulture, University of Agronomic Sciences and Veterinary Medicine of Bucharest, 59 Marasti Blvd., 011464 Bucharest, Romania

**Keywords:** hydroxyapatite, clam shells wastes, methylene blue degradation, photocatalysis, environmental remediation

## Abstract

This work aims to evaluate the potential use of natural wastes (in particular, clam shells) to synthesize one of the most well-known and versatile materials from the phosphate mineral group, hydroxyapatite (HAP). The obtained material was characterized in terms of morphology and composition using several analytical methods (scanning electron microscopy—SEM, X-ray diffraction—XRD, X-ray fluorescence—XRF, Fourier transform infrared spectroscopy—FTIR, thermal analysis—TGA, and evaluation of the porosity and specific surface characteristics by the Brunauer–Emmett–Teller—BET method) in order to confirm the successful synthesis of the material and to evaluate the presence of potential secondary phases. The developed material was further doped with iron oxide (HAP-Fe) using a microwave-assisted method, and both materials were evaluated in terms of photocatalytic activity determined by the photodecomposition of methylene blue (MB) which served as a contaminant model. The best results (approx. 33% MB degradation efficiency, after 120 min. of exposure) were obtained for the hydroxyapatite material, superior to the HAP-Fe composite (approx. 27%). The utilization of hydroxyapatite obtained from clam shells underscores the importance of sustainable and eco-friendly practices in materials syntheses. By repurposing waste materials from the seafood industry, we not only reduce environmental impact, but also create a valuable resource with diverse applications, contributing to advancements in both healthcare and environmental protection.

## 1. Introduction

Along with the human demands of growth and rapid industrialization, countless efforts have been made to address the issue of hazardous contaminants from industrial wastewater, from detection to identifying new methods for their removal [1,2]. Recycling wastewater demands significant human effort, given that the need for clean water has almost doubled every twenty years [3].

Methylene blue (MB), a frequently used azo dye for cotton, wood, and silk textiles, can cause eye burns that may lead to permanent damage to the eyes of humans and animals. Inhalation of MB can lead to respiratory issues, while ingestion can cause a burning sensation, nausea, vomiting, sweating, and profuse cold sweats [4].

In the context of the circular economy and considering the need to address the issue of underused biowastes, the development of new added-value materials using such wastes as raw materials emerges as a promising solution [5].

Over the past few years, photocatalysts have gained essential status in green chemistry, and these developments are anticipated to boost the adoption of catalysts [6], due to their ease of recyclability, and the straightforwardness of product separation [7]. The preference for natural-based photocatalysts is also influenced by their eco-friendly nature [8], as well as by the low costs associated with the process [9].

Hydroxyapatite (HAP), as a naturally occurring wide-bandgap semiconductor with a hydrophilic nature, exhibits a surface with a strong tendency to adsorb various macromolecules [10], being a commonly used material in adsorption-based de-pollution technologies. The photocatalytic activity of this type of material is explained by the transformation of phosphate groups into highly reactive hydroxyl and superoxide radicals under UV irradiation, which leads to the decomposition of non-degradable azo dyes [11]. Several studies present the possibility of developing HAP by processing a wide variety of calcium-rich resources, such as eggshells, clams or mussel shells, corals or other seashells, or even animal bones [12,13].

Clam shells are considered calcium-rich wastes [14], often underused [15]; by a controlled process of calcination and subsequent treatments, these wastes can be used for the obtaining of hydroxyapatite biomaterials [16], which, in turn, can have a broad spectrum of applications, ranging from biomedicine to environmental remediation [17,18].

As a continuously evolving wastewater treatment method, photocatalysis is steadily garnering increased attention for its capability to completely mineralize various compounds, making it a potentially significant application for HAP. Semi-conductors such as titanium dioxide [19,20] and zinc oxide [21], are considered the most active photocatalysts, having as their main disadvantage the high costs involved in their production and application [22]. According to the available literature data, HAP has been applied as a catalyst for the degradation of organic pollutants, with promising results [23]. For example, Valizadeh et al. synthesized a magnetite-hydroxyapatite composite used for the degradation of Acid Blue 25 dye, obtaining an 80% decomposition degree after 90 min [24]. Other works presented a 75% efficiency of methylene blue degradation after 250 min, using synthetic hydroxyapatite [25].

In this context, this work aimed to study the possibility of developing hydroxyapatite from natural resources, specifically from the shells of the striped Venus clam (one of the most encountered clam species in the Black Sea), as well as to evaluate its photocatalytic properties, for the degradation of a well-known model azo dye, namely methylene blue (MB).

## 2. Materials and Methods

### 2.1. Synthesis Methods

The biowastes, considered calcium oxide precursors, were represented by the shells of striped Venus clams (*Chamelea gallina* Linnaeus, 1758, *Veneridae*). The clam shells were collected from the Navodari beach area, Constanta County, Romania, in May–June 2023. After species identification, the shells were washed, disinfected, and ground, until particle diameters ≤ 0.5 mm were obtained (as determined by sieving).

The grounded sample was calcined at 1000 °C, the obtained material (calcium oxide) being used for the synthesis process, following a recipe previously presented by our group [26,27], using (NH_4_)_2_HPO_4_ (Merck KGaA, Darmstadt, Germany). Briefly, the two reagents (as-obtained calcium oxide and the phosphate solution) were separately dissolved in distilled water; the calcium-containing solution was heated to 80 °C, and the phosphate solution (with the pH adjusted to 10) was drop-added to the calcium solution under vigorous stirring. The reaction, performed at a molar ratio Ca solution to P solution = 2:1, was further performed at 80 °C and a pH = 10 [28], under continuous stirring; the pH regulator used was NaOH (Chimreactiv, Bucharest, Romania). After 3 h of reaction, the precipitated material was repeatedly washed with bi-distilled water (until a neutral pH was recorded), filtered, and rinsed with ethanol (Chimreactiv, Bucharest, Romania), the final hydroxyapatite material being obtained by vacuum drying the ethanol-containing gel at 45 °C.

**Figure 1:**
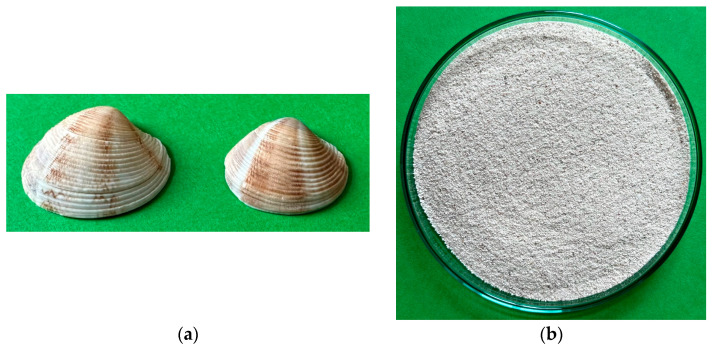
The striped Venus clam shells used in the study: representative samples of the raw material (**a**) and powder used for the studies (**b**).

Figure 1 presents the striped clams chosen for the experiments and, also, the powder obtained after the grinding procedure.

In order to obtain the iron-oxide decorated HAP, the hydroxyapatite material (0.1 g), ferric chloride (Merck KGaA, Darmstadt, Germany, 0.15 g) used as metal oxide precursor and sodium carbonate (Merck KGaA, Darmstadt, Germany, 0.25 g) were dissolved in 5 mL of ethylene glycol (Merck KGaA, Darmstadt, Germany), following a recipe previously described by our group [29]. The resulting mixture was subjected to a reaction for 10 min in a microwave flow reactor (Discover 2.0 Microwave Flow) at a temperature of approximately 160 °C and 300 W power. Thereby, the solutions were mixed using stirring systems, followed by a filtration step, and finally drying to obtain the sample encoded as HAP-Fe. The selection of the metal used for decoration was performed considering the results previously obtained by our group [29], which suggest that the use of iron oxide could enhance the photocatalytic properties of the final composite.

### 2.2. Analytical Characterization

The diffractograms were recorded using a Rigaku SmartLab 9 kW diffractometer (Rigaku Corporation, Tokyo, Japan), using the following operating parameters: 2θ/θ scan mode, scanning range: 5 to 90 degrees (2θ), Cu K_α_ radiation (λ = 1.54059 Å), at 45 kV and 200 mA; the identification of the present phases was achieved by comparison with the ICDD database, using the dedicated data interpretation software PDXL (v. 2.7.2.0.).

The crystallite size was determined using the Debye–Scherrer equation:

(1) $$Dp=\frac{\left(K\times \lambda \right)}{\left(\beta \times cos\theta \right)}$$

where Dp = the average size of the crystallites,

K—the Scherrer constant (for cubic structures, K =0.94),
β = represents the width at half-height of the diffraction maximum,
θ = the Bragg angle,
λ = the wavelength—1.54059 Å, in our case.

For the calculation of the crystallinity degree of the hydroxyapatite phases, the following equation [30] was used:

(2) $$CD={\left(\frac{0.24}{\beta \left(002\right)}\right)}^{3}$$

where β(002) represents the full width at half maximum (FWHM) of (002) reflections.

The elemental composition of the samples was determined using a surface non-destructive method, X-ray fluorescence (XRF), using a portable XRF analyzer (Vanta C Series Handheld XRF) equipped with an X-ray tube of 40 kV, rhodium anode and Silicon Drift detector. XRF measurements were performed in GeoChem mode, applying two different energy beams of 60 s each, in order to quantify heavy and respectively light elements.

To study the functional groups of the obtained photocatalyst, Fourier transform infrared spectroscopy (FTIR) analyses were performed in the range of 400–4000 cm^−1^, using a Jasco FTIR 6300 spectrometer (Jasco Corporation, Tokyo, Japan).

A thermogravimetric analyzer TGA Q5000IR (TA Instruments, New Castle, DE, USA) was used for establishing the thermal stability of the samples, in the temperature range 25–700 °C. The following parameters were selected: heating rate 10 °C/min and synthetic air flow of 50 mL/min.

The particle size and the surface morphology of the materials, but also their elemental compositional analysis, were studied with the help of a field emission scanning electron microscope Hitachi TM4000plus II (SEM, Tokyo, Japan), equipped with an energy dispersive spectroscopy accessory (EDX). The analyses were performed following the technical operating parameters: back-scattering electrons detector (BSE), acceleration voltage: 10–15 kV, resolution range between ×500 and ×2000.

The porosity and specific surface characteristics of the hydroxyapatite material were determined by the Brunauer–Emmett–Teller (BET) method, measuring the N_2_ adsorption-desorption isotherms at −196 °C, in a Quantachrome Nova 2200e automated gas adsorption system (Quantachrome Instruments Corporate Drive, Boynton Beach, FL, USA). The samples were previously degassed at 100 °C for 3 h, in order to determine the specific surface area (S_BET_) as well as the total pore volumes (V_total_).

The OriginPro 2022b (version 9.9.5.167) data analysis software was used to process and graphically display the obtained results.

### 2.3. Photodegradation of Model Dye Contaminant

The photocatalytic activity of the obtained materials was evaluated by the photodecomposition of a model dye, namely methylene blue (MB), usually found in textile wastewater. To determine the photocatalytic properties of the synthesized materials, paint at a total weight of 3 g containing 90% styrene-acrylic film-forming material and 10% apatitic material was prepared, following a procedure previously presented by our group [31]. The paint was processed using an automatic pigment processing machine (J. Engelsmann AG, Ludwigshafen am Rhein, Germany) [32] and then deposited on the glass slides to dry for 48 h, in a layer approximately 50 ± 1 µm thick. To study the dye degradation under natural conditions, a Xenon lamp (Atlas Xenotest 150S+, Atlas Material Testing Solutions, Mount Prospect, IL, USA) was used, the dried glass slides were immersed in vessels containing 30 mL of a 1 g/L solution of the pollutant, and underwent the irradiation process for 120 min, following an internal method developed based on standard methods [33,34].

UV-vis reflectance spectra on the coatings were recorded at an exposure time of 0, 30, 60, 90, and 120 min, using a Jasco V570 spectrometer (Jasco Corporation, Tokyo, Japan) equipped as a reference with a 150 mm Spectralon integrating sphere (Jasco ILN-472).

The following equation was used to calculate the photodegradation efficiency of the materials:

(3) $$DE=\frac{\left(R_{t}-R_{0}\right)}{R_{t}}\times 100$$

where:

DE = photodegradation efficiency (%),
R_t_ = initial reflectance of the coating,
R_0_ = reflectance of the coating at a certain exposure time to light.

## 3. Results and Discussions

### 3.1. Characterization of the Obtained Materials

**Figure 2:**
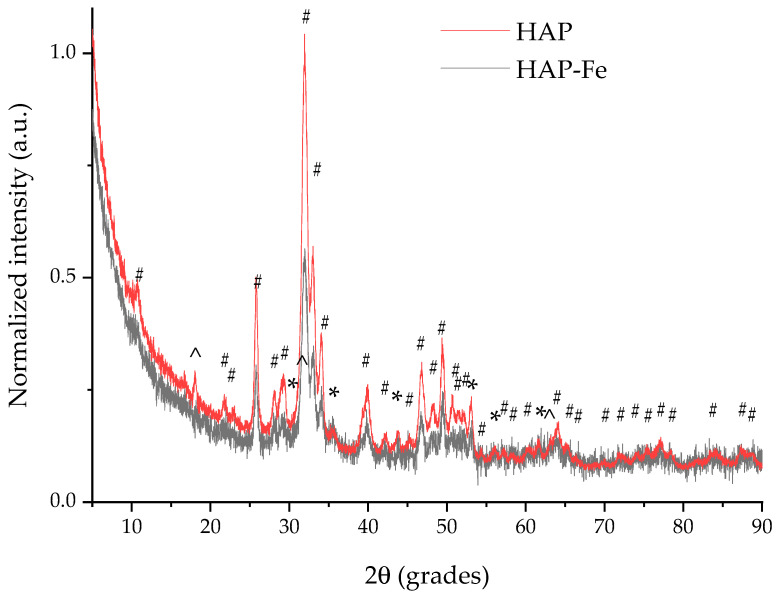
X-ray diffractogram of biosynthesized-HAP (HAP) and HAP-Fe, where # = peaks corresponding to hydroxyapatite; * = magnetite; ^ = nahpoite; attribution of major XRD peaks is presented in Table 1, while the attribution of minor peaks is presented in the Supplementary Material, Table S1.

The obtained materials were characterized by X-ray diffraction in order to confirm the obtaining of the envisaged materials. The recorded XRD patterns (Figure 2 presenting the normalized X-ray diffractograms) revealed the presence of the characteristic peaks of hydroxyapatite: 25.8 degrees, which correspond to the (002) plane, 31.93 degrees—(211), 32.9 degrees—(300), 34.02 degrees—(202), 39.9 degrees—(130), 46.7 degrees—(222), and 49.4 degrees—(2,1,3), identified by comparison with the ICDD card no. 01-073-8417. From Figure 2 and Table S1, the presence of a secondary phase, nahpoite (Na_2_(HPO_4_)), can also be observed, the most intense peak associated with its presence being the one observed at 18.05 degrees (2ϴ), which corresponds to the (002) plane (identification performed by comparison with the ICDD card no. 01-086-0606), a phenomenon explained by the use of sodium hydroxide as pH regulator during the synthesis. Other peaks of the nahpoite phase overlap those corresponding to the hydroxyapatite phase.

The synthesis of HAP-Fe was confirmed by the increase in the intensity of several peaks, due to the contribution of the magnetite (Fe_3_O_4_) phase, apparent for the major XRD peaks at: 35.6 degrees, corresponding to the (311) plane, 37.1 degrees—(222), 46.78—(331), 53.2 degrees—(422), and 74.16—(533), respectively, alongside some other minor peaks (as presented in the Supplementary Material, Table S1); the identification of the magnetite phase was made by comparison with the ICDD card no. 01-079-0417.

Very interestingly, the XRD diffractogram of the composite material does not clearly exhibit the characteristic nahpoite peak (around 18°, 2ϴ), which could be an indicator of the transformation of the secondary phase during iron oxide formation. In contrast to the HAP diffractogram, HAP-Fe presented considerably lower intensities of the peaks and a lower signal-to-noise ratio, a characteristic attributed to the presence of the magnetite phase [35], which would suggest the presence of the magnetite particles on the surface of the sample. Table 1 presents the assignment of the major XRD peaks (relative intensity ≥ 15% of the intensity of the most intense peak) observed in the two diffractograms, while the attribution of the minor peaks is presented in the Supplementary Material, Table S1. At the same time, the increase in the intensity of several peaks (presented in Table 1) can be directly attributed to the contribution of the magnetite phase, an increase which led them to be assigned as “major peaks” (i.e., peaks no. 7, 10, 12, and 14). Alongside the appearance of some other minor magnetite-specific peaks at 30.56 degrees, corresponding to the (220) plane, 37.08 degrees—(222), and 62.38 degrees—(440), an increase in the intensity of some minor peaks overlapping the major hydroxyapatite phase is also observed, for the peaks at 57.19—(511), 65.26—(531), and 66.66—(442), however without a sufficient contribution to transform them into “major peaks”.

Using Equation (1) applied to the most intense peak (corresponding to the diffraction plane (211)), it was possible to determine the crystallite size of the hydroxyapatite phase in the HAP material (10.72 nm), and for the HAP-Fe composite (10.59 nm). The very small variation in crystallite size would suggest that the microwave formation of the iron compounds indeed represented a decoration of the hydroxyapatite, and not a change in the nature of the material, such as the replacement of Ca^2+^, which would be accompanied by a modification in unit cell dimensions.

The crystallinity degree, calculated according to Equation (2), showed a medium crystallinity degree, with no significant differences between the two samples (58% for HAP, and 59% for HAP-Fe).

The elemental composition of the photocatalysts is presented in Table 2, as determined by XRF.

The XRF analysis illustrated that HAP-Fe showed a slow decrease in the Ca content compared to the simple HAP, probably due to uneven distribution in the analyzed sample, while, in the HAP-Fe sample, the presence of Fe content was proven, at approximately 20.31%.

**Figure 3:**
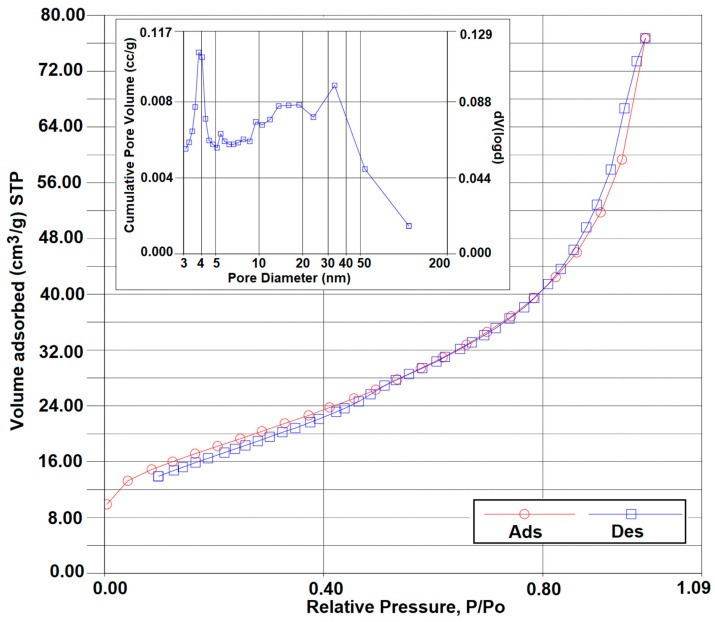
Nitrogen adsorption/desorption isotherms of HAP; inset—BJH pore size distribution function.

Using the Brunauer–Emmett–Teller (BET) method, relevant information was obtained regarding the porosity and specific surface characteristics of the hydroxyapatite material (nitrogen adsorption/desorption isotherms and Barrett–Joyner–Halenda pore size distribution presented in Figure 3, while other parameters are presented in Table 3).

According to the International Union of Pure and Applied Chemistry which classifies pores into micropores (<2 nm diameter), mesopores (2–50 nm diameter), and macropores (>50 nm diameter), based on the average pore diameter obtained (~7 nm), the sample can be classified as mesoporous. By comparing the results obtained with those presented by other works (Table 3), it can be noticed that the specific surface and pore diameter are superior to several examples from the literature data. At the same time, by comparison with the data published by our group regarding HAP obtained by the same method (although using commercial chemical reagents) [38], a reduction in both specific surface and pore parameters can be noticed.

**Figure 4:**
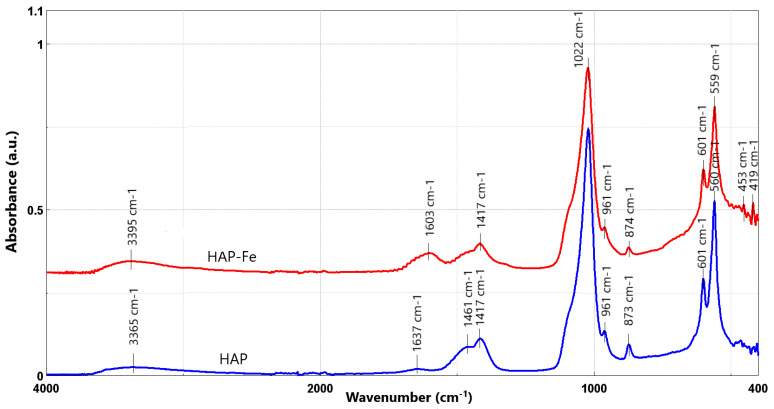
FT-IR spectra obtained for the developed materials: HAP (blue) and HAP-Fe (red).

Figure 4 presents the FT-IR spectra of the two obtained materials.

For both materials, the peaks characteristic to apatitic materials are presented, namely: 1022, 961, and 561 cm^−1^, respectively. In the HAP FTIR spectrum, the main peak for the phosphate vibration modes can be observed at 1022 cm^−1^, due to its symmetric υ3 vibration [26,27,28,29]. The peaks, present at 3365 cm^−1^ for simple HAP and 3395 cm^−1^ for HAP-Fe, mark the stretch of the hydroxyl groups, while the peaks shown at 1417 and 1637 cm^−1^ can be correlated with the vibration of carbonate groups [39]. Even if HAP does not contain any carbonate phases, according to the X-ray diffractograms presented before, the peaks displayed in the FT-IR spectra may be observed due to unreacted products due to the synthesis. Another explanation could be their very high transmittance, making it possible to observe their presence even at lower concentrations.

The characteristic peaks for the magnetite phase, which is present in the HAP-Fe material, are observed at 3395, 1603, and, respectively, 601 cm^−1^ [40]. The ν2 vibrations of the Fe-O bonds could be marked by the appearance of the bands at 453 and 419 cm^−1^ [41,42], even if this phenomenon took place at higher wavenumbers, in comparison with the literature data [43]. All the other signals appearing in the spectrum are distinctive for hydroxyapatite.

**Figure 5:**
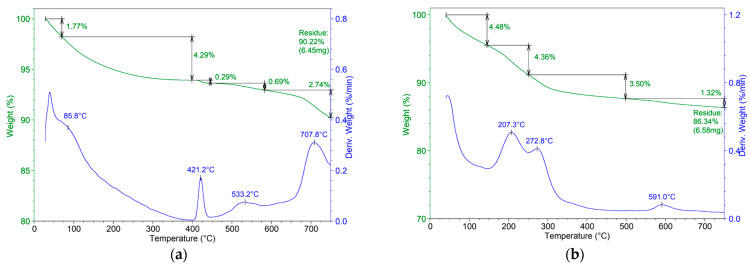
Thermogravimetric analyses for the obtained materials: (**a**) HAP and (**b**) HAP-Fe.

The thermal behavior of the samples, presented in Figure 5a,b, was also studied from room temperature to 700 ℃, in order to assess the stability of the HAP-Fe composite, in comparison with the simple HAP. The obtained results at 700 ℃ revealed a residue of 90.22% in the case of HAP and 86,34% for HAP-Fe. The first stage of weight loss was recorded at temperatures below 120 °C, of approximately 1.77% for HAP and 4.48% for HAP-Fe, which can be attributed to the desorption of surface-bound water [44]. The mass loss recorded from up to 400 °C recorded for the HAP sample (4.29%) can be explained by the loss of structural water, similar to the mass loss recorded for the HAP-Fe sample up to 250 °C (4.36%) [45]. Also, at approximately 421 ℃, another phenomenon appeared in the case of HAP, which can be related to the transformation of nahpoite into the more stable tetrasodium pyrophosphate, which represents the final product after heating disodium phosphate to 450 °C [46,47]. After 500 °C, the process of apatite dehydroxylation represents the main thermal event [29] (0.69%), while the mass loss of 2.74% registered for HAP after approx. 600 °C, could be assigned to the decomposition of the pyrophosphate or the decomposition of other minor compounds formed during the synthesis process. Regarding the HAP-Fe composite, a mass loss at higher temperatures (1.32% for HAP-Fe) can be observed, which can be assigned to the supplementary contribution of the oxidation reactions of magnetite into hematite [48], besides the dehydroxylation process. On the other hand, in the thermogram of the HAP-Fe composite, no thermal event associated with the transformation of nahpoite is observed. This could be explained by the formation of a more stable phase during the microwave synthesis of the iron oxide phase, which could not be identified by X-ray diffraction, due to the many XRD peaks of the hydroxyapatite. However, this aspect should be further explored and elucidated in future studies.

**Figure 6:**
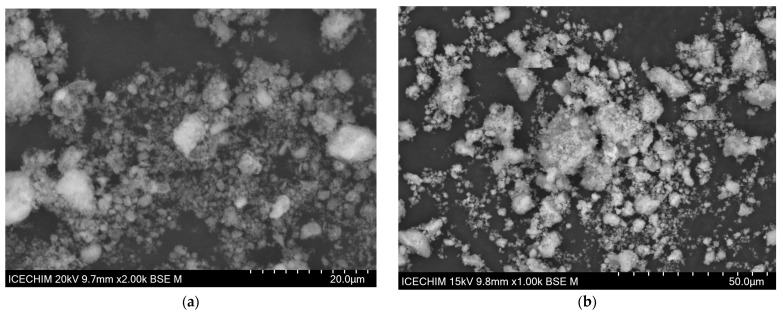

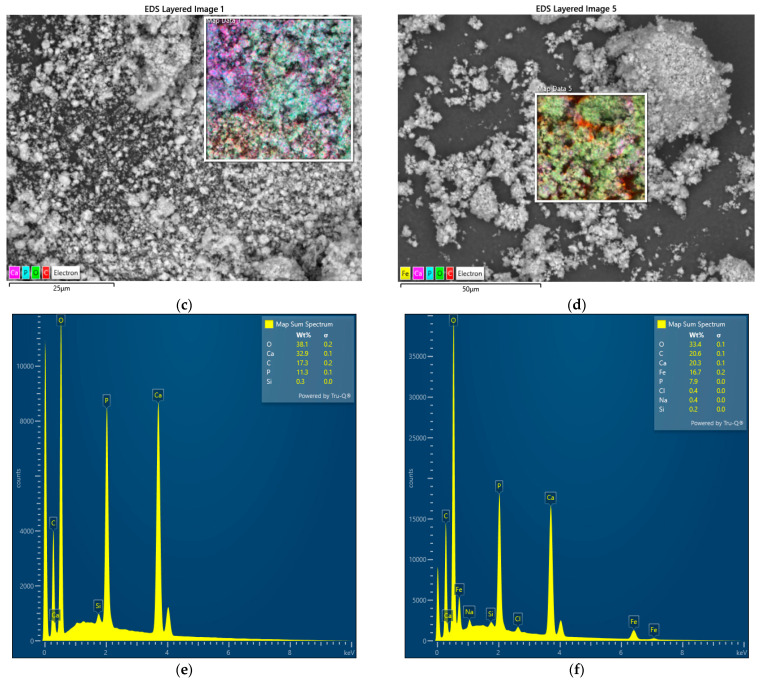
SEM images of the obtained materials: (**a**) HAP; (**b**) HAP-Fe; elemental distribution map: (**c**) HAP; (**d**) HAP-Fe; EDX spectra: (**e**) HAP, (**f**) HAP-Fe.

Figure 6a,b presents the SEM images obtained for the synthesized materials, Figure 6c,d presents the element distribution map, while Figure 6e,f shows the EDX spectra recorded. Lower magnification images are also presented in the Supplementary Material, Figure S2.

SEM images revealed the formation of larger circular/spherical particle agglomerations, generally homogenous in size and morphology. From SEM images, no significant variations in the apparent morphology can be identified for the two samples; also, the absence of elongated (needle-like) particles is visible; the synthesis path followed apparently favors the appearance of spherical ones. The recorded EDX spectra also demonstrates the presence of iron in the composite material (Figure 6f). The presence of main composition elements confirmed the formation of the material and the decoration of the material.

### 3.2. Studies on the Methylene Blue Photodegradation

The photocatalytic activity was evaluated by the degradation of methylene blue in a photocatalytic reactor under visible light irradiation by using a UV filter to block the UV light.

**Figure 7:**
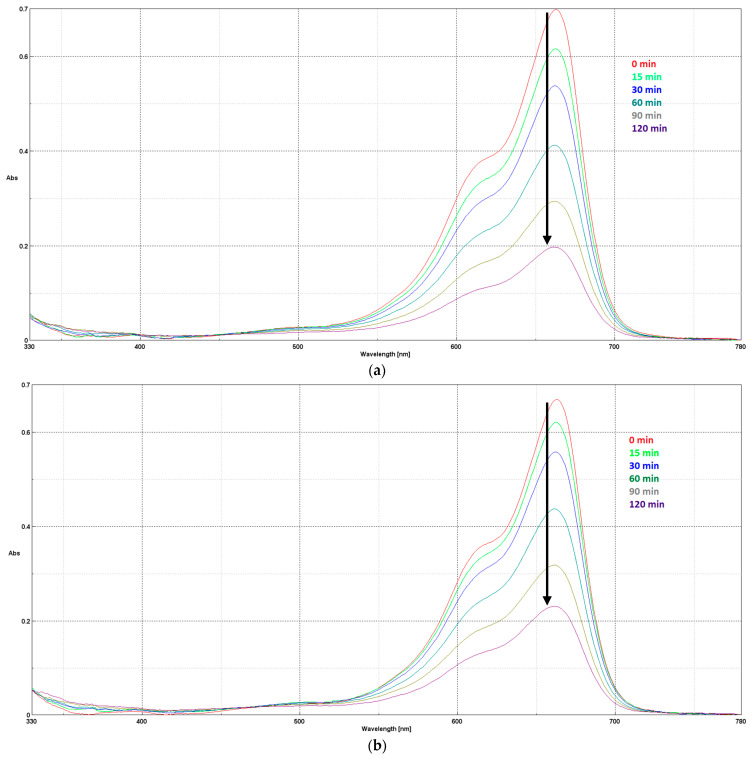
MB photodecomposition in time (0–120 min) using coatings containing: (**a**) HAP; (**b**) HAP-Fe.

The UV absorption spectra (Figure 7) show the photocatalytic decomposition of methylene blue, the decrease in absorbance suggesting the decrease in its concentration in the solution, being recorded over time (0–120 min). The absorption spectra of MB using HAP and HAP-Fe show a distinct band at 664 nm, attributed to the auxochromic group of MB [49]. The decrease in the intensity of this peak (as seen in Figure 7a,b) is also correlated with the degradation of MB.

**Figure 8:**
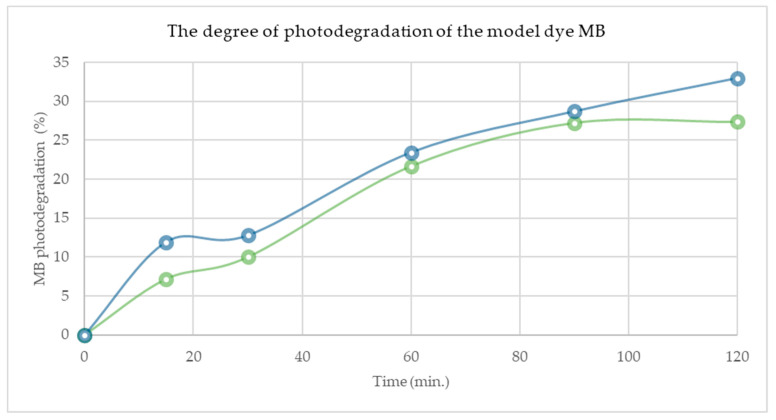
Photodecomposition degree of methylene blue using coatings containing HAP (blue) and HAP-Fe (green), t = 0–120 min.

From the graph presented in Figure 8 it can be concluded that the obtained degree of photodecomposition after 120 min of exposure is approximately 33% in the case of HAP and 27% in the case of HAP-Fe; also, for the composite HAP-Fe, the MB photodegradation appears to reach a plateau after 90 min., for the HAP material it follows a continuously increasing trend, which would suggest that, after a longer exposure time, the degree of photodegradation of MB could be enhanced.

**Figure 9:**
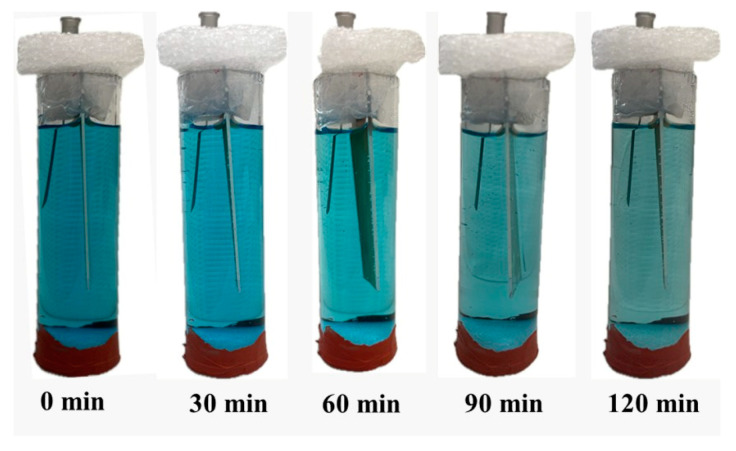
Evolution of Methylene Blue photodegradation over time (0–120 min) in the case of using HAP coatings.

Figure 9 presents the visual difference in terms of the color of the MB solution after 120 min of the photodecomposition process.

By comparing the results obtained in this study with literature data (Table 4), it can be noticed that the MB photodegradation efficiency of the synthesized materials is much lower than the one recorded for composites constructed using HAP as support material [50,51,52], for which photodegradation efficiencies > 50% are usually reached. On the other hand, when compared with literature data of simple HAP, obtained by the use of synthetic reagents [53], a superior MB photodegradation efficiency can be observed; also, the efficiency recorded is slightly lower than HAP obtained by the use of similar biowastes (mussel shells) [16], however, in this case, HAP was subjected to thermal treatment and the irradiation was performed for a much longer period. The efficiency of HAP-Fe, although lower than expected, was still higher than that recorded by other authors for pure HAP [53].

**Figure 10:**
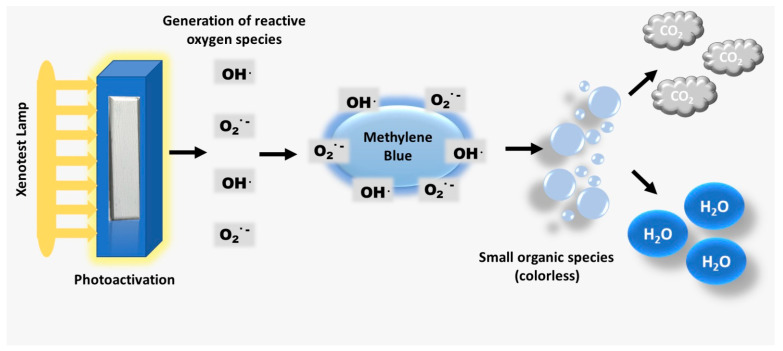
Schematic representation of the MB photocatalytic degradation process induced by HAP/HAP-Fe materials.

The proposed mechanism for the photodegradation of MB in the presence of HAP and HAP-Fe is presented in Equations (4)–(8) and Figure 10.

(4) $$O_{2}+e^{-}\to O_{2}^{\cdot }$$

(5) $$O_{2}^{\cdot }+H^{+}\to {HO}_{2}^{\cdot }+{OH}^{\cdot }$$

(6) $${2OH}^{\cdot }\to H_{2}O_{2}$$

(7) $$H_{2}O_{2}+e^{-}\to {OH}^{\cdot }+{OH}^{-}$$

(8) $$MB+{HO}_{2}^{\cdot }+{OH}^{\cdot }+O_{2}^{\cdot }\to MB\;degradation\;products$$

In conclusion, it has been demonstrated that the clam shell-derived HAP can be used as a photocatalyst for the degradation of aqueous pollutants, especially azo-dyes, with methylene blue being successfully degraded. At the same time, the efficiency of MB photodegradation was not enhanced by the presence of the magnetite phase in the apatitic material, the process needs further studies in order to elucidate all the aspects involved in the process.

## 4. Conclusions

The study demonstrated that clam shell waste (striped Venus) represents an alternative approach to creating valuable resources with diverse applications while sustaining the bioeconomy, through the valorization of natural wastes. Two materials were developed, bio-derived hydroxyapatite and a hydroxyapatite/magnetite composite, which were characterized through several modern analytical techniques to assess the obtainment of desired properties while also confirming their phase composition. The materials underwent the photocatalytic process with the goal of obtaining the photodecomposition of a model azo dye, namely methylene blue. The results have shown a 33% degree of photodegradation of MB in the case of HAP which was slightly higher than in the case of HAP-Fe.

The major difference between the literature data and this work is represented by the formulation of the proposed materials as photoactive paints. The photocatalytic properties of the developed paints (even at the relatively low concentration proposed in this work) represents a potential source for developing active paints and coatings, which can lead to the in situ improvement of environment quality.

This de-pollution technology represents important progress for environmental protection, offering highly efficient materials with low costs involved, using easy logistics, with the primary condition of using customized optimized materials, being also considered an “eco-friendly” synthesis method.

## Figures and Tables

**Table 1:** Attribution of the major peaks present in the diffractograms of the HAP and HAP-Fe samples. H—hydroxyapatite, N—nahpoite, M—magnetite; the most intense peak, used for the differentiation of the peaks, is marked with yellow color.

Peak No.	HAP			HAP-Fe		
	Position (2ϴ)	d-Value	Attribution	Position (2ϴ)	d-Value	Attribution
1	10.7254	8.24196	H(1,0,0)	10.83	8.16	H(1,0,0)
2	25.803	3.450	H(0,0,2), N(0,2,0)	25.77	3.454	H(0,0,2), N(0,2,0)
3				29.09	3.067	H(2,1,0)
4	31.937	2.8000	H(2,1,1), N(0,2,2)	31.939	2.7998	H(2,1,1), N(0,2,2)
5	32.982	2.7136	H(3,0,0), N(1,2,−2)	33.007	2.7116	H(3,0,0), N(2,0,−2)
6	34.024	2.6329	H(2,0,2), N(1,1,2)	34.05	2.6309	H(2,0,2)
7				35.611	2.51907	H(3,0,1), N(1,1,−4), M(3,1,1)
8	39.90	2.2576	H(1,3,0), N(0,1,4)	39.87	2.259	H(1,3,0), N(0,3,1)
9				42.129	2.14316	H(1,3,1), N(1,2,−4)
10	46.728	1.9424	H(2,2,2), N(1,3,1)	46.78	1.940	H(2,2,2), N(2,2,−4), M(3,3,1)
11	49.400	1.8434	H(2,1,3), N(1,2,−5)	49.49	1.8404	H(2,1,3), N(1,2,−5)
12				53.203	1.72024	H(0,0,4), N(2,1,−6), M(4,2,2)
13				63.88	1.456	H(5,0,2), N(1,3,4)
14				74.159	1.27759	H(5,2,1), N(1,2,6), M(5,3,3)
15				84.322	1.1476	H(0,0,6), N(2,2, −9)
16				88.738	1.10156	H(2,0,6), N(4,2,−8)

**Table 2:** Elemental analysis of the HAP and HAP-Fe samples.

Elements	HAP (%)	HAP-Fe (%)
P	21.48 ± 0.20	21.32 ± 0.20
Ca	41.83 ± 0.64	38.80 ± 0.56
Fe	0	20.31 ± 0.32
Light elements	34.07 ± 0.82	19.10 ± 1.00

**Table 3:** Comparative evaluation of the specific surface area and pore diameter obtained in this study with relevant literature data.

Sample	S_BET_ [m^2^/g]	S_BJH_ [m^2^/g]	V_total_ [cm^3^/g]	D_medium pore_ [nm]	D_BJH_ [nm]	Ref.
HAP from biowastes	64.94	41.00	0.1187	7.31	3.82	Current work
HAP (chemical reagents)	30.68	-	-	-	-	[36]
HAP (chemical reagents)	62.7	71.2	0.38	-	1.6	[37]
HAP (chemical reagents)	72.4	-	0.367	-	9.568	[38]

**Table 4:** Comparative evaluation of the photodegradation efficiency of the materials obtained in this study with relevant literature data.

Sample	Material Formulation	Photodegradation Experimental Parameters	MB Photodegradation Efficiency (%)	Ref.
Copper-doped hydroxyapatite encapsulated into polycaprolactone nanofibrous	Nanofibrous membranes	Visible light source (500 watts), 70 min.	94.3	[50]
Eggshell derived HAP nanoparticles entrenched on two-dimensional g-C_3_N_4_ nanosheets	Composite prepared by adding C_3_N_4_ into the HAP reaction mixture	Photocatalyst added directly into the dye solution, visible light source (500 watts), 100 min.	93.69	[51]
Spherical porous HAP-supported zinc oxide	ZnO loaded on HAP obtained using synthetic reagents	Photocatalyst added directly into the dye solution, irradiation with black light for 120 min (wavelength: 368 nm, UV intensity: ~240 µW/cm^2^).	58	[52]
Gold loaded HAP nanoparticles	Gold loaded onto HAP Obtained using synthetic reagents by a microwave method (0.055 wt% Au)	Photocatalyst added directly into the dye solution, white light 10 W xenon lamp irradiation, 9 h.	32.47	[53]
HAP	Obtained using synthetic reagents	Photocatalyst added directly into the dye solution, white light 10 W xenon lamp irradiation, 9 h.	25	[53]
HAP obtained using green lipped mussel shells	Heat treated HAP	Photocatalyst added directly into the dye solution, irradiation with UV light (at 254 nm), no oxygen added in the solution, 6 h	39	[16]
HAP	HAP obtained using striped Venus clam shells	The photocatalytic activity of the obtained materials HAP/HAP-Fe at 10% concentration mixed in paint and deposited on glass slides; irradiation using natural conditions, exposure time 120 min	33	Present work
HAP-Fe	HAP decorated with magnetite using a microwave method		27	Present work

## Data Availability

The raw data supporting the conclusions of this article will be made available by the authors on request.

## Supplementary Materials

The following supporting information can be downloaded at: https://www.mdpi.com/article/10.3390/ma17112492/s1, Figure S1: Comparative view of the XRD patterns obtained for the synthesized materials and ICDD entries used for the identification of phases present: (a) HAP, (b) HAP-Fe; Table S1: Attribution of the minor peaks (relative intensity < 15% of the most intense peak) present in the diffractograms of the HAP and HAP-Fe samples. H—hydroxyapatite, N—nahpoite, M—magnetite; Figure S2: SEM images of the obtained materials, at different magnifications: (a) and (b) HAP; (c) and (d) HAP-Fe.
