# Supplementary material for: Clam Shell-Derived Hydroxyapatite: A Green Approach for the Photocatalytic Degradation of a Model Pollutant from the Textile Industry
Source: Materials (Basel). 2024 May 22;17(11):2492. doi: 10.3390/ma17112492 (PMC11172594; doi:10.3390/ma17112492)
Supplement: Supplementary file 1 [file materials-17-02492-s001.zip › materials-2997597-supplementary.pdf]

Supplementary Material

Clam Shell-Derived Hydroxyapatite: A Green Approach for the Photocatalytic Degradation of a Model Pollutant from the Textile Industry

Roxana Ioana Matei (Brazdis) [1,2,†], Anda Maria Baroi [1,3], Toma Fistos [1,2], Irina Fierascu [1,3], Maria Grapin [1,2,†], Valentin Raditoiu [1], Florentina Monica Raduly [1], Cristian Andi Nicolae [1] and Radu Claudiu Fierascu [1,2,*]

[1] National Institute for Research & Development in Chemistry and Petrochemistry—ICECHIM Bucharest,
202 Spl. Independentei, 060021 Bucharest, Romania; roxana.brazdis@icechim.ro (R.I.M.); anda.baroi@icechim.ro (A.M.B.); toma.fistos@icechim.ro (T.F.); irina.fierascu@icechim.ro (I.F.);
maria.grapin@icechim.ro (M.G.); vraditoiu@icechim.ro (V.R.); monica.raduly@icechim.ro (F.M.R.);
cristian.nicolae@icechim.ro (C.A.N.)

[2] Faculty of Chemical Engineering and Biotechnologies, National University of Science and Technology Politehnica Bucharest, 1-7 Gh. Polizu Str., 011061 Bucharest, Romania

[3] Faculty of Horticulture, University of Agronomic Sciences and Veterinary Medicine of Bucharest, 59 Marasti Blvd., 011464 Bucharest, Romania

* Correspondence: fierascu.radu@icechim.ro

† These authors contributed equally to this work, being considered the main authors of the study.

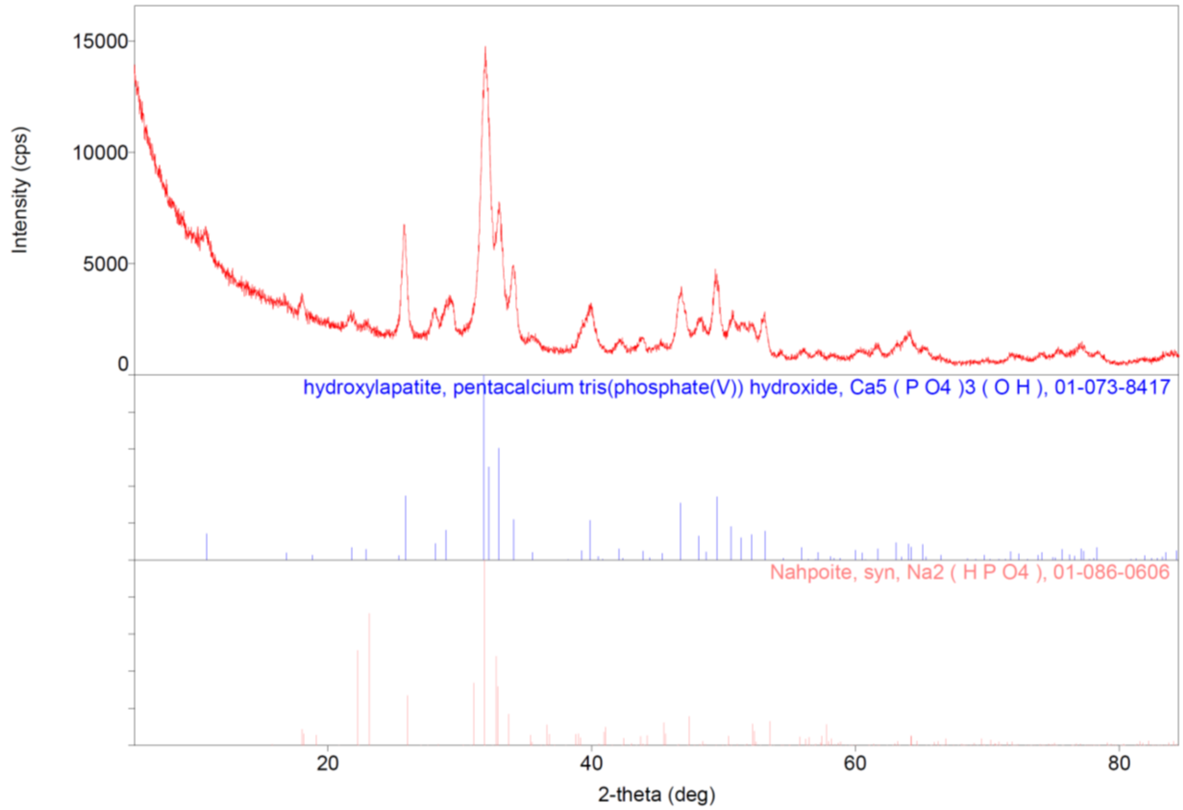

Intensity (cps)
15000
10000
5000
0
hydroxylapatite, pentacalcium tris(phosphate(V)) hydroxide, Ca5 (P O4)3 (O H), 01-073-8417
Nahpoite, syn, Na2 (H P O4), 01-086-0606
20
40
60
80
2-theta (deg)

(a)

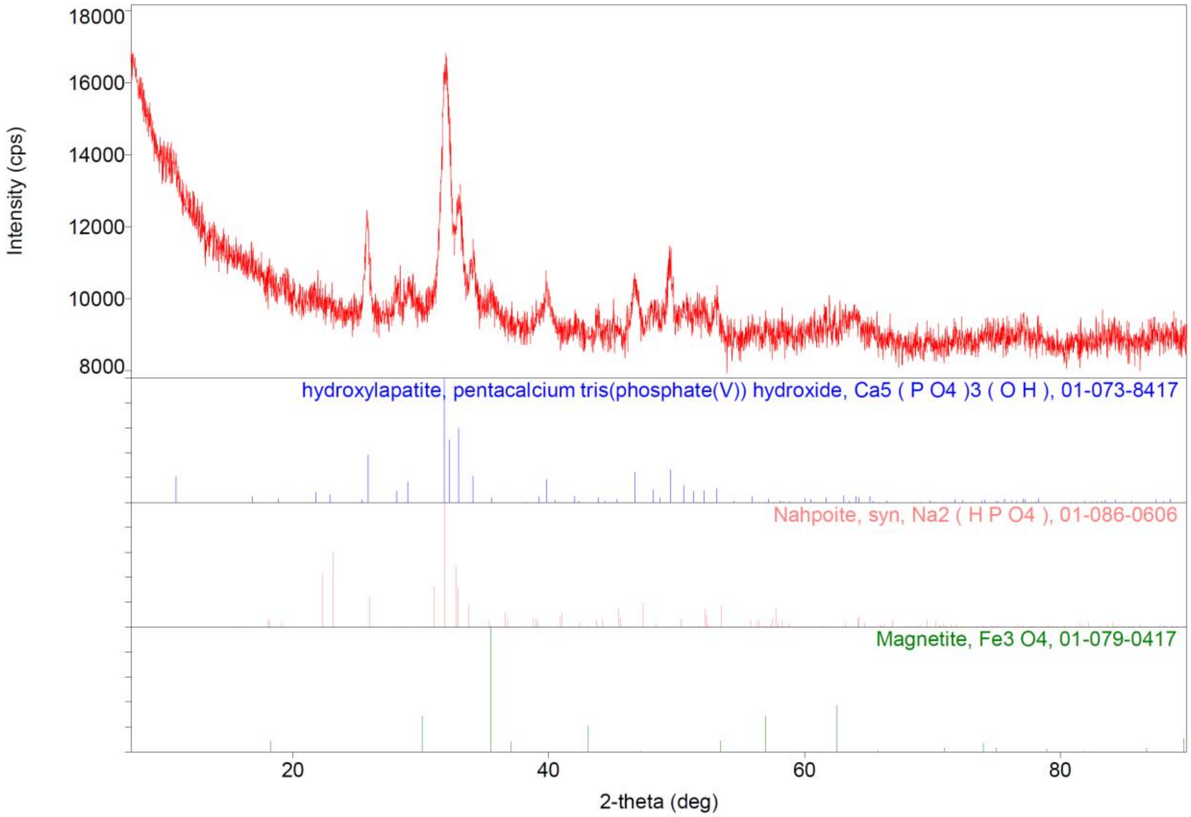

Intensity (cps)
18000
16000
14000
12000
10000
8000
hydroxylapatite, pentacalcium tris(phosphate(V)) hydroxide, Ca5 (P O4)3 (O H), 01-073-8417
Nahpoite, syn, Na2 (H P O4), 01-086-0606
Magnetite, Fe3 O4, 01-079-0417
20
40
60
80
2-theta (deg)

(b)

Figure S1. Comparative view of the XRD patterns obtained for the synthesized materials and ICDD entries used for the identification of phases present: a) HAP, b) HAP-Fe.

Table S1. Attribution of the minor peaks (relative intensity <15% of the most intense peak) present in the diffractograms of the HAP and HAP-Fe samples. H – hydroxyapatite, N – nahpoite, M – magnetite;

**Peak no.**	**HAP**			**HAP-Fe**		
	Position (2θ)	d-value	Attribution	Position (2θ)	d-value	Attribution
1	16.7661	5.28359	H(1,0,1)			
2	18.05	4.911	N(0,0,2)			
3				19.2801	4.59993	H(1,1,0), N(1,0,−2)
4	21.87	4.061	H(2,0,0), N(0,1,2)	21.9435	4.04726	H(2,0,0), N(0,1,2)
5	22.8814	3.88346	H(1,1,1), N(1,1,0)			
6	28.11	3.172	H(1,0,2), N(0,2,1)	28.1815	3.16398	H(1,0,2), N(0,2,1)
7	29.24	3.052	H(2,1,0)			
8				30.5645	2.9225	M(2,2,0), N(0,1,3)
9	35.45	2.530	H(3,0,1), N(1,1,−4)			
10				37.08	2.4224	M(2,2,2), N(0,0,4)
11	40.86	2.204	H(2,2,1), N(1,2,2)	40.91	2.20414	H(2,2,1), N(1,2,2)
12	42.19	2.1400	H(1,3,1), N(1,2,−4)			
13				42.2694	2.13638	H(3,0,2), N(1,3,−1)
14	43.79	2.0655	H(1,1,3), N(0,3,2)	43.9515	2.05844	H(1,1,3), N(0,3,2)
15				45.143	2.00684	H(2,0,3), N(0,2,4)
16	48.20	1.8863	H(1,3,2), N(1,2,3)	48.297	1.88289	H(1,3,2), N(0,1,5)
17	50.58	1.8030	H(2,3,1), N(2,2,1)	50.5399	1.80447	H(3,2,1), N(2,1,2)
18	51.56	1.7713	H(1,4,0), N(2,0,−6)			
19				52.0117	1.75681	H(4,1,0), N(2,0,−6)
20	52.24	1.7496	H(3,0,3), N(3,1,−3)			
21	52.995	1.7265	H(0,0,4), N(2,3,−1)			
22	55.98	1.641	H(2,2,3), N(1,2,4)	56.2171	1.63495	H(2,2,3), N(0,0,6)
23				57.1983	1.60921	H(1,1,4), N(1,4,−2),

24				58.2497	1.58265	M(5,1,1) H(2,0,4), N(3,1,0)
25	60.2	1.535	H(4,2,0), N(1,1,5)	60.0019	1.54055	H(4,2,0), N(3,2,−1)
26	61.51	1.5063	H(2,1,4), N(0,4,3)	61.684	1.50251	H(1,2,4), N(2,1,−7)
27				62.3849	1.4873	M(4,4,0), N(2,2,3),
28	63.78	1.4580	H(5,0,2), N(0,3,5)			
29	65.2407	1.42894	H(1,5,1), N(2,4,−3)	65.2586	1.42859	H(1,5,1), N(2,4,−3), M(5,3,1)
30	66.34	1.4080	H(1,4,3), N(3,3,−2)	66.6603	1.40191	H(1,4,3), N(1,2,−7), M(4,4,2)
31				68.8331	1.36286	H(3,1,4), N(2,0,−8)
32				69.7443	1.34727	H(1,5,2), N(3,1,2)
33	71.94	1.3114	H(4,0,4), N(1,5,0)	71.8469	1.31292	H(1,1,5), N(1,5,−2)
34				72.8983	1.29656	H(5,2,0), N(0,2,7)
35	74.13	1.2780	H(2,5,1), N(2,3,−7)			
36	75.3831	1.25987	H(3,2,4), N(4,1,−6)	75.3514	1.26032	H(3,2,4), N(2,1,5), M(6,2,2)
37	77.09	1.2362	H(4,3,2), N(3,3,1)	76.9634	1.23789	H(4,3,2), N(3,4,−2)
38				78.225	1.22105	H(3,0,5), N(4,0,0), M(4,4,4)
39	78.41	1.2187	H(1,4,4), N(3,1,3)			
40				81.2389	1.18319	H(2,2,5), N(2,4,3), M(7,1,1)
41	83.73	1.1542	H(6,0,3), N(4,3,−4)	83.1313	1.161	H(6,0,3), N(3,1,−9)
42	87.31	1.1158	H(1,1,6), N(4,3,−1)	87.3366	1.11559	H(1,1,6), N(1,6,−1), M(6,4,2)
43	88.47	1.1042	H(2,6,1), N(0,6,2)			

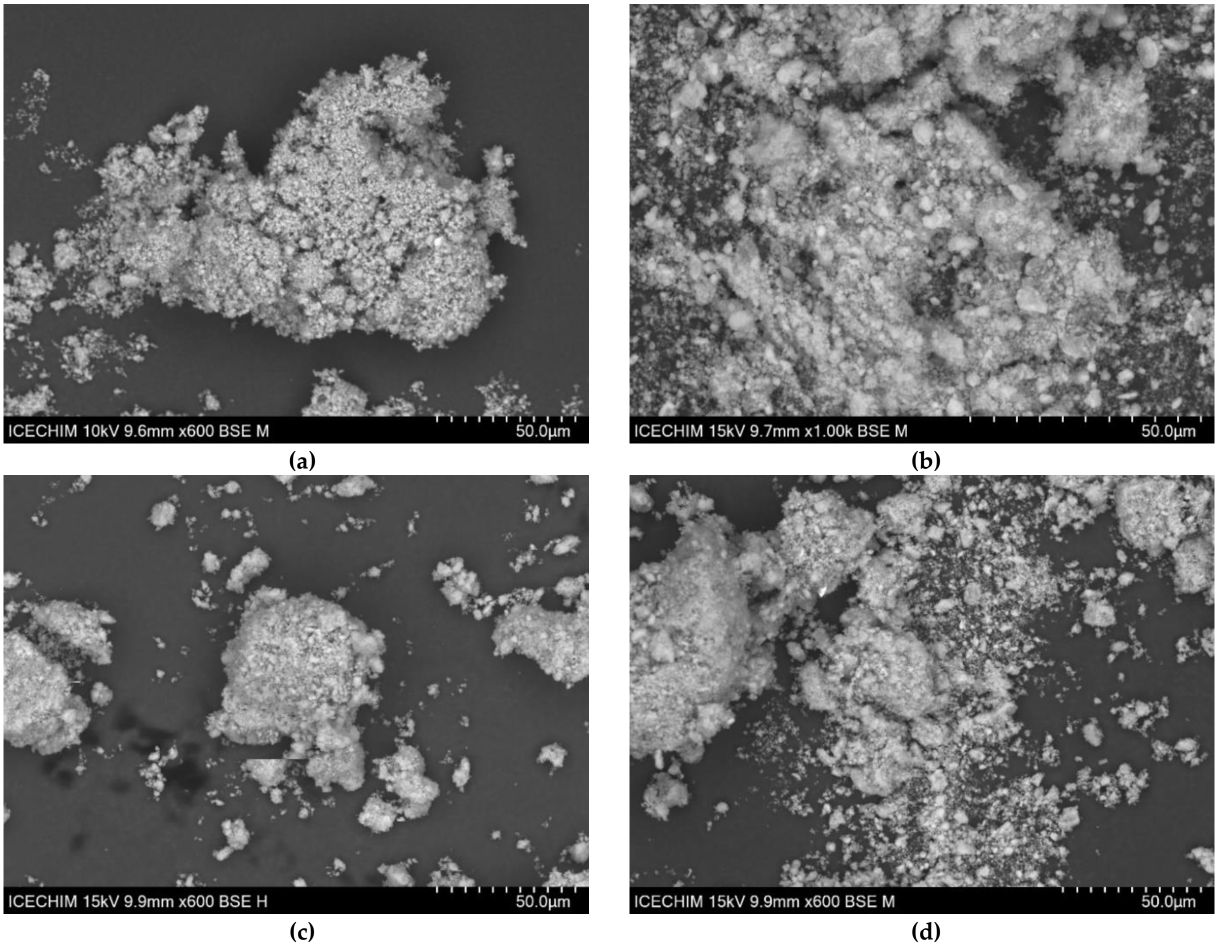

Figure S2. SEM images of the obtained materials, at different magnifications: a) and b) HAP; c) and d) HAP-Fe
